# The CMV-encoded G protein-coupled receptors M33 and US28 play pleiotropic roles in immune evasion and alter host T cell responses

**DOI:** 10.3389/fimmu.2022.1047299

**Published:** 2022-12-07

**Authors:** Timothy M. White, Cassandra M. Bonavita, Brent A. Stanfield, Helen E. Farrell, Nicholas J. Davis-Poynter, Rhonda D. Cardin

**Affiliations:** ^1^ Department of Pathobiological Sciences, Louisiana State University, Baton Rouge, LA, United States; ^2^ School of Chemistry and Molecular Bioscience, University of Queensland, Brisbane, QLD, Australia; ^3^ Child Health Research Centre, University of Queensland, Brisbane, QLD, Australia

**Keywords:** Cytomegalovirus (CMV), viral latency, T cell memory, memory inflation, Viral G protein-coupled receptor

## Abstract

**Introduction:**

Human cytomegalovirus (HCMV) is a global health threat due to its ubiquity and lifelong persistence in infected people. During latency, host CD8^+^ T cell responses to HCMV continue to increase in a phenomenon known as memory inflation. We used murine CMV (MCMV) as a model for HCMV to characterize the memory inflation response to wild-type MCMV (KP) and a latency-defective mutant (ΔM33_stop_), which lacks M33, an MCMV chemokine receptor homolog. M33 is essential for normal reactivation from latency and this was leveraged to determine whether reactivation in vivo contributes to T cell memory inflation.

**Methods:**

Mice were infected with wild-type or mutant MCMV and T cell responses were analyzed by flow cytometry at acute and latent time points. Ex vivo reactivation and cytotoxicity assays were carried out to further investigate immunity and virus replication. Quantitative reverse-transcriptase polymerase chain reaction (q-RTPCR) was used to examine gene expression during reactivation. MHC expression on infected cells was analyzed by flow cytometry. Finally, T cells were depleted from latently-infected B cell-deficient mice to examine the in vivo difference in reactivation between wild-type and ΔM33_stop_.

**Results:**

We found that ΔM33_stop_ triggers memory inflation specific for peptides derived from the immediate-early protein IE1 but not the early protein m164, in contrast to wild-type MCMV. During ex vivo reactivation, gene expression in DM33stop-infected lung tissues was delayed compared to wild-type virus. Normal gene expression was partially rescued by substitution of the HCMV US28 open reading frame in place of the M33 gene. *In vivo* depletion of T cells in immunoglobulin heavy chain-knockout mice resulted in reactivation of wild-type MCMV, but not ΔM33_stop_, confirming the role of M33 during reactivation from latency. Further, we found that M33 induces isotype-specific downregulation of MHC class I on the cell surface suggesting previously unappreciated roles in immune evasion.

**Discussion:**

Our results indicate that M33 is more polyfunctional than previously appreciated. In addition to its role in reactivation, which had been previously described, we found that M33 alters viral gene expression, host T cell memory inflation, and MHC class I expression. US28 was able to partially complement most functions of M33, suggesting that its role in HCMV infection may be similarly pleotropic.

## Introduction

Human cytomegalovirus (HCMV), a member of the betaherpesvirinae family, is a ubiquitous and usually nonlethal viral pathogen, which nonetheless causes considerable morbidity and mortality in neonates, transplant recipients, and immunosuppressed populations (1). HCMV, like other herpesviruses, undergoes an acute phase of lytic replication followed by lifelong latency. Acute infection in immunocompetent individuals is usually mild and latency is asymptomatic, although latent infection may be associated with increased morbidity of autoimmune, cardiovascular, and neoplastic diseases (2, 3). Immunosuppressive conditions such as organ transplant, chemotherapy, and AIDS, lead to HCMV reactivation from latency and local or systemic lytic infection (4–6). Latent HCMV is also clinically relevant during aging, when long-term changes to the activation status and proliferative capacity of immune cells result in altered responses to vaccines and infections (7–10). However, ascribing positive or negative impacts on the immune system to HCMV is difficult, and indeed both may occur (7–10). HCMV is species-specific and therefore cannot be studied in an animal model. Murine CMV (MCMV), a closely related betaherpesvirus that infects mice, shares many homologous genes and provides a useful tool for *in vivo* experiments. MCMV infection in mice is biphasic, reactivates during immunosuppression, and produces similar cell-mediated immune responses to HCMV-infected humans (11–15).

The immune responses to both HCMV and MCMV are complex, involving early activation of innate defenses, in particular innate lymphoid cells (ILCs) and natural killer (NK) cells, which secrete interferon-γ and provide direct cell-mediated cytotoxicity (16). Within a few days of the initiation of infection, intervention of the adaptive immune system, primarily CD8^+^ TCRαβ^+^ T cells, leads to suppression of viral replication (17). During latency, CD8^+^ T cells (CTLs) maintain constant immune surveillance for viral antigens and prevent systemic reactivation (17).

Unlike most pathogens, both MCMV and HCMV trigger long-term oligoclonal proliferation of viral epitope-specific CD8^+^ T cells, termed ‘memory inflation’ (14). Eventually, these T cell clones may represent a substantial fraction of total CTLs, far more than any other individual T cell clone (14). The functional capacity of inflationary CTLs remains a topic of debate (18, 19). Despite high surface expression of killer cell lectin-like receptor G1 (KLRG1), an inhibitory marker classically associated with senescence, inflationary CTLs are sufficient to prevent lethal MCMV infection (20). *Ex vivo* analyses demonstrate that inflationary CTLs remain fully capable of cytokine secretion and antigen-mediated cytotoxicity, and they do not express most other lymphocyte exhaustion markers (20). The mechanisms driving T cell memory inflation have been extensively studied but remain incompletely described (13, 21–23). Like other herpesviruses, HCMV and MCMV express transcripts during latency (TELs), which are RNA transcripts detectable in the absence of replicating virus (24, 25). TELs may be translated into proteins, which are then proteolytically digested and presented on MHC class I, thus priming epitope-specific CTLs (reviewed extensively in (13)). Alternatively, memory inflation may be triggered by sporadic reactivation events, during which a viral episome in a latently-infected cell spontaneously enters the replicative cycle, inducing focal lytic replication and corresponding immune cell interactions. In BALB/c mice, two protein epitopes are the primary drivers of memory inflation: one from immediate-early protein 1 (IE1; YPHFMPTNL) (26), and one from the m164 protein (AGPPRYSRI) (27).

CMVs also encode numerous proteins that aid in immune evasion and reactivation (3). Among these are several G protein coupled receptors (GPCRs) which closely mimic mammalian C-C chemokine receptors and are known as viral chemokine receptor homologs (vCKRs) (28, 29). vCKRs have a broad array of functions, including constitutive signaling, reactivation from latency, and chemokine sequestration (29–34). HCMV encodes four vCKRS: US27, US28, UL33, and UL78. MCMV encodes only two: M33 and M78. M33, the first MCMV GPCR to be described, plays a role in replication in the salivary gland and in viral latency (30, 32). Although UL33 and M33 are positional homologs in the HCMV and MCMV genomes, respectively, M33 has been shown to more closely mirror the function of US28, with roles in constitutive signaling and reactivation from latency (30–33). M33 has also been shown to enhance migration of infected cells *in vivo*, promoting dissemination of the virus (33). However, whether vCKRs impact T cell memory responses is unknown.

In this report, the interplay between vCKRs and T cell responses to acute and latent MCMV infection is investigated. M33-deficient MCMV demonstrates impaired reactivation, which can be leveraged *in vivo* to dissect the mechanisms that drive memory inflation during latent infection (30). In the current studies, we use analysis of inflationary epitope-specific CD8^+^ T cells to elucidate whether M33 expression impacts immune responses during latency.

## Methods

### Cell culture

NIH 3T3 fibroblast cells (Cell Line Services #400103, Eppelheim, Germany) were grown in complete medium consisting of Dulbecco’s modified Eagle medium (DMEM) with 4.5 g/L glucose and L-glutamine without sodium pyruvate (Corning, USA) supplemented with 10% fetal bovine serum (FBS; Hyclone, Logan, USA), 0.3% sodium bicarbonate, 4 mM HEPES, 2 mM L-glutamine, and 1x gentamycin solution. Cells were incubated in 5% CO_2_ at 37°C. SV40-immortalized mouse salivary gland mesenchymal cells [mSGM cells (35)] were maintained in complete medium as above with the following modifications: FBS concentration was lowered to 5% and an additional 50µM G418 (Gibco) was added to maintain selection.

### Viruses and virus stock preparation

Viruses used in this study were K181 Perth (KP), ΔM33_stop_, and ΔM33-US28^+^ (See **Supplemental Table 1**). Tissue culture virus stock was produced by inoculating NIH 3T3 fibroblasts (Cell Line Services #400103) at MOI of 0.005 with salivary gland-derived virus (KP) or plaque purified virus from original cultures (ΔM33_stop_, ΔM33-US28^+^). At 6-8 days post infection, stocks were collected and concentrated by centrifugation. Pellets were collected and sonicated to free intracellular viral particles. When necessary, virus stocks were additionally purified by ultracentrifugation on 20% sucrose cushions to minimize contamination with cellular debris.

### Plaque assays

Plaque assays were performed using NIH 3T3 cells as previously described (30). Briefly, serial 10-fold dilutions of samples were placed on sub-confluent NIH 3T3 cell monolayers in 12-well plates (Corning) and incubated at 37° and 5% CO2 for 1 h. Supernatant was then removed and overlaid with 0.75% w/v carboxymethylcellulose (Thermo Fisher, Waltham, MA) in modified Eagle medium (Gibco) with 10% FBS, 2mM L-glutamine, 0.5x NEAA solution (Gibco), and 1x gentamicin solution (Gibco). Plates were monitored for plaque formation and fixed with methanol at 5-6 dpi. Cell monolayers were then stained with modified Giemsa stain (Sigma-Aldrich, St. Louis, MO) for 1 hour. Plaques were counted visually using an inverted microscope.

### *In vivo* studies

For *in vivo* infections, 5-week-old female BALB/c ByJ (Jackson Laboratory, Bar Harbor, USA) or 5-week-old female immunoglobulin heavy chain-knockout BALB/c mice (*C.Cg-Igh-J^tm1Dhu^*; Taconic Biosciences, Germantown, USA) were used. At appropriate endpoints, mice were euthanized by intraperitoneal injection of tribromoethanol in *tert*-amyl alcohol at a 1:40 dilution in PBS (2.5% Avertin). Mice were kept under specific pathogen-free conditions at the Louisiana State University Department of Laboratory Animal Medicine. All mouse protocols were approved by the Institutional Animal Care and Use Committee at Louisiana State University.

### Explant reactivation assays

Explant assays were carried out on tissues from latently infected mice as previously described (30). Briefly, tissues were divided into 3 equal portions, each in a well of a 6-well tissue culture plate (Corning). 4 ml of complete medium was added to each well and tissues were minced into 2-mm^3^ fragments. Plates were then incubated at 37°C and 5% CO_2_. At the indicated time points, supernatant was collected, using care not to aspirate tissue fragments. The supernatant was then replaced with fresh medium. Supernatants from explants were then sonicated and titered by plaque assay.

### Flow cytometry

Single-cell suspensions of lymphocytes were prepared from spleens by gently homogenizing tissues in phosphate buffered saline (PBS) or complete medium, followed by straining through a 40-µm cell strainer. Erythrocytes were lysed using RBC lysis buffer (Beckton Dickinson, Franklin Lakes, USA) and remaining nucleated cells were resuspended at 10^6^-10^7^ cells/ml in PBS with 2% FBS. Fc receptor block was performed using monoclonal αCD16/CD32 for 10 minutes prior to staining (Beckton Dickinson). Extracellular staining was conducted on live cells for 45-60 minutes at 4°C, after which cells were fixed with 2% formaldehyde. When indicated, intracellular staining was conducted by permeabilizing with BD Perm/Wash Buffer (Beckton Dickinson) prior to incubation with antibodies at the appropriate dilution. For salivary gland and lung lymphocyte isolation, tissues were minced and incubated at 37°C in collagenase type I (ThermoFisher, Waltham, MA, USA) or collagenase type II (ThermoFisher), respectively, at a concentration of 100 U/ml in Hank’s balanced salt solution for 4 hours, followed by homogenization and straining with a 40-µm cell strainer. The cells were then carefully layered on Lympholyte M (Cedarlane Laboratories Ltd., Canada) and centrifuged at 500x g for 20 minutes. Lymphocytes were collected from the liquid interface and filtered again through a 40-µm strainer prior to staining. Flow cytometry was performed on lymphocytes within 24 hours after staining and fixation using a Fortessa X-20 flow cytometer (Beckton Dickinson) and results were analyzed using FlowJo software (Beckton Dickinson).

### Antibodies and tetramers

Antibodies used were αCD3-FITC, αCD4-PerCP-Cy5.5, αCD8-BV711, αCD69-BV421, αCD107a-BV786, αCD127-BV510, αKLRG1-BV650, αIFNγ-PE-CF594, αIFNγ-AF700, αCD19-APC, αCD3-BV711, or αCD8-BV510 (Beckton Dickinson) and αH-2K^d^-AF488, αH-2L^d^-PE, or αH-2D^d^-AF648, and matched isotype controls (BioLegend, San Diego, USA). For all antibodies, a dilution of 1:100 to 1:250 was found to produce optimal results. MHC tetramers were produced by the NIH Tetramer Core Facility at Emory (Atlanta, USA). Tetramers were H-2L^d^ IE1 (YPHFMPTNL)-PE and H-2D^d^ m164 (AGPPRYSRI)-APC. Tetramer staining was conducted at a dilution of 1:500 to 1:1000 on live cells simultaneous with antibody staining. Dead cells were excluded by staining with BD Horizon fixable viability stain at 450 or 520 emission (Beckton Dickinson).

### *In vivo* T cell depletion

For *in vivo* reactivation studies, 5-week-old female immunoglobulin heavy chain-knockout BALB/c mice (*C.Cg-Igh-J^tm1Dhu^*; Taconic Biosciences) were inoculated intraperitoneally with 10^6^ PFU of KP or ΔM33_stop_. After latency was established (70 dpi) T cell were depleted as previously described (6). Briefly, mice were injected IP with 500μg of αCD4 (clone GK1.4) and αCD8 (clone 2.43). At day 5 and day 10 post initiation of depletion, mice were given an additional 250μg IP. Mice were sacrificed at day 14 post depletion start. Efficacy of depletion was confirmed by flow cytometry (**Figure S10**).

### T cell isolation and cytotoxicity

For T cell cytotoxicity assays, T cells were isolated from splenocytes using MACS pan-T cell isolation kit (Miltenyi Biotec, Cambridge, USA) according to manufacturer’s instructions. MACS-enriched T cells and mSGM salivary gland cells were cocultured at various effector-target ratios in complete medium containing RPMI (Gibco) supplemented with 10% FBS and 1x penicillin/streptomycin (Gibco).

### Cytotoxicity and apoptosis quantification

Cytotoxicity was measured by monitoring for caspase activation using BioTracker NucView 488 caspase substrate (Biotium, Fremont, CA), according to manufacturers’ instructions, using a final concentration of 1.25 µM in complete RPMI. All cytotoxicity and apoptosis assays were carried out on an Incucyte machine (Agilent, Santa Clara, CA) and analyzed with Incucyte software (Agilent). Any fluorescent cells with area less than 300 µm^2^ were excluded from analysis to avoid counting apoptotic T cells or cell debris.

### Gene sequencing and validation

To confirm the presence of the in-frame stop codons in the ΔM33_stop_ genome, a 544 base pair segment of the M33 gene spanning the stop codon insert site was amplified using a Platinum Taq PCR kit (ThermoFisher, Waltham, USA), purified by gel electrophoresis on a 2% agarose gel and visualized using ethidium bromide. The band was excised from the gel and purified using a Wizard SV Gel and PCR Clean-up System (Promega, Madison, USA) and the amplicon was sequenced by Sanger sequencing using a Big Dye Terminator 3.1 system (Applied Biosystems, Foster City, USA). Alignment was performed using Benchling (Biology Software, San Francisco, USA).

### DNA quantitative polymerase chain reaction

Viral genome copy numbers were quantified using qPCR and the viral gene E1 normalized to the host gene glyceraldehyde-3-phosphate dehydrogenase (GAPDH). Primers are shown in **Supplemental Table 2**. qPCR was conducted using SYBR Green ROX Mastermix Fast (Qiagen, Hilden, Germany) according to manufacturer’s instructions on an AB7000 machine (Applied Biosystems, Foster City, USA). A temperature profile of 95°C for 10 min followed by cycles of 95°C for 15 s and 60°C for 1 min. qPCR was run for 40 cycles and cycle threshold (CT) values of >35 were considered to be negative. Viral genome copies per cellular genome were then calculated with the assumption of diploid cells. Studies were conducted with a minimum of 5 biological replicates and 3 technical replicates each.

### Reverse transcriptase qPCR

RNA transcripts were quantified using qRT-PCR. Briefly, RNA was purified from tissues or cells using Qiagen RNeasy kit (Qiagen, Hilden, Germany) according to manufacturer’s instructions, with an additional on-column DNase I digestion step (ThermoFisher). cDNA was transcribed from 100 ng total RNA using an RT2 cDNA kit (Qiagen) according to manufacturer’s instructions. qPCR was conducted using SYBR Green ROX Mastermix Fast (Qiagen, Hilden, Germany) according to manufacturer’s instructions on an AB7000 machine (Applied Biosystems, Foster City, USA). A temperature profile of 95°C for 10 min followed by cycles of 95°C for 15 s and 60°C for 1 min. qPCR was run for 40 cycles and cycle threshold (CT) values of >35 were considered to be negative. Experiments had a minimum of 3 biological replicates and 3 technical replicates each. Primers are shown in **Supplemental Table 2**.

### Statistical analysis

All statistical analysis was computed using either FlowJo (Beckton Dickinson, Franklin, USA) or Prism 9 (GraphPad, San Diego, USA). Figures are labeled with the statistical tests used; in most cases, ANOVA with Tukey’s *post-hoc* test was used after testing for normality. Significance is shown as *, p<0.05; **, p<0.01; ***, p<0.001; ****, p<0.0001.

## Results

### ΔM33 MCMV has a normal replicative phenotype in the lungs and spleen during acute infection

**Figure 1:**
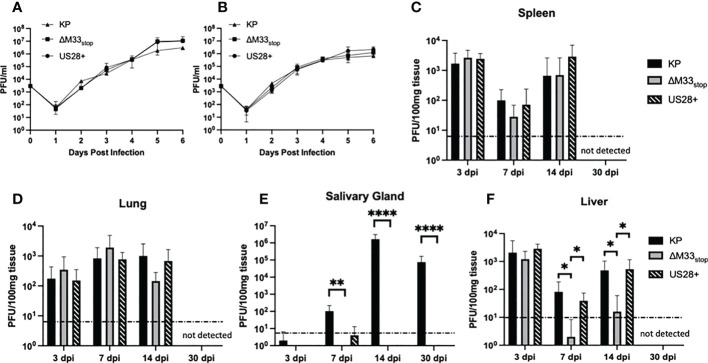
Growth kinetics of KP, ΔM33_stop,_ and US28+ MCMV are similar *in vitro* and in most tissues *in vivo*. **(A, B)** mSGM cells **(A)** or NIH 3T3 cells **(B)** were infected at an MOI of.01 and total virus was titered at 24 hour intervals (n=6). **(C-F)** BALB/c ByJ mice were inoculated I.P. with 1x10^6^ PFU of KP, ΔM33_stop,_ or US28^+^ MCMV and sacrificed at 3, 7, 14, and 30 days post infection. Viral kinetics are shown in the spleen **(C)**, lungs **(D)**, salivary gland **(E)**, and liver **(F)**. Dotted line represents the limit of detection. Data presented are mean ± sd. Mouse experiments were conducted three times (3, 7, and 14 dpi; n=15) or once (30 dpi; n=5). Comparisons represent two-way ANOVA with significance indicated as: *, p<0.05; **, p<0.01; ****, p<0.0001.

An M33-knockout virus, ΔM33_stop_, which contains an 11-base pair insertion containing a premature stop codon in the M33 open reading frame was used for these studies. Disruption of the M33 ORF was verified by Sanger sequencing (**Figure S1**) (32). The truncated protein produced by this ORF lacks the extracellular ligand-binding domain and the signaling motif required for interaction with the Gα subunit of the G protein pathway, precluding both ligand-dependent and constitutive signaling in addition to preventing interaction with or sequestration of chemokines (33). A second mutant virus, ΔM33-US28+ (hereafter referred to as US28+) in which the M33 ORF is replaced with the HCMV US28 gene, was also used. Wild-type KP, ΔM33_stop,_ and US28^+^ MCMV were grown in mSGM salivary gland mesenchymal-like cells (35) (**Figure 1A**) and NIH 3T3 fibroblasts (**Figure 1B**). All three viruses replicated to similar levels and no significant difference in growth was detected in either mutant as compared with KP (p>0.1).

To determine growth characteristics *in vivo*, mice were inoculated intraperitoneally with KP, ΔM33_stop,_ or US28^+^. At 3, 7, 14, and 30 days post infection (dpi), mice were sacrificed and virus replication in tissues was determined by plaque assay. The ΔM33_stop_ virus reached titers similar to KP in the lung (**Figure 1C**) and spleen (**Figure 1D**), in contrast to results previously reported for ΔM33_BT2_, which contains a *LacZ* expression cassette in the M33 locus (30, 32). As described previously, ΔM33_stop_ and US28^+^ were unable to replicate in the salivary gland, however, KP reached a high titer that persisted to 30 dpi (**Figure 1E**) (32). Interestingly, in the liver, a tissue in which natural killer (NK) cells are primarily responsible for eliminating MCMV infection, we found that ΔM33_stop_ was controlled by 7 dpi in most animals, in contrast to US28^+^ and KP (**Figure 1F**; p<0.05) (36). Virus titers in all tissues but the salivary glands were below the limit of detection at 30 dpi, indicating normal immune clearance.

### ΔM33_stop_ reactivates *ex vivo* with diminished efficiency

**Figure 2:**
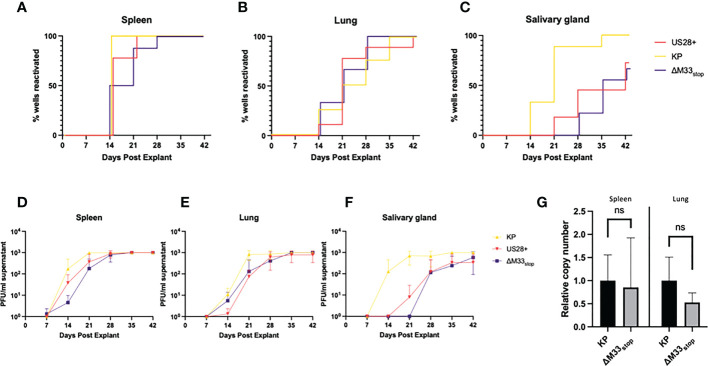
KP and ΔM33_stop_ MCMV reactivation ex vivo. Mice were infected I.P. with 10^6^ PFU of KP, US28^+^, or ΔM33_stop_ MCMV. At 70 dpi, mice were sacrificed and tissues were explanted. **(A-C)** US28^+^ (red line), ΔM33_stop_ (purple line) and KP (gold line) are shown in the spleen **(A)**, lung **(B)**, and salivary gland **(C)**. **(D-F)** Titers of virus from explant supernatant in the spleen **(D)**, lung **(E)**, and salivary gland **(F)** shown in PFU/ml. **(G)** Viral genome copy number, normalized to cell genome number, shown relative to wild-type KP (p>0.2). Data represent 5 animals, with each tissue divided into 3 wells (A-F; n=5) or 3 animals **(G)**. Statistical test used in **(G)** is Student T test. Statistics shown as: ns, not significant.

Both the viral GPCRs US28 and M33 have been shown to play crucial roles in reactivation from latency (30, 33, 37). Most of these studies used ΔM33_BT2_, a virus in which the M33 ORF is disrupted by the *Escherichia coli* β-galactosidase gene (*LacZ*), allowing for colorimetric staining (30, 38). Since the β-galactosidase enzyme itself is known to be immunogenic (39), the ΔM33_stop_ mutant was used in these studies instead. To characterize reactivation of the ΔM33_stop_ virus, explant assays were performed, confirming the M33-associated latency defect. Mice infected with KP, ΔM33_stop_, or US28^+^ MCMV were sacrificed at 70 days post infection and tissues were cultured *ex vivo* as previously described (30, 40). Reactivation from these explanted tissues was assessed weekly by plaque assay. Consistent with our expectations, wild-type KP reactivated from nearly 100% of tissues and from all mice in the study. ΔM33_stop_ reactivated from the spleen, but with significantly reduced efficiency and from fewer tissue fragments compared to KP using Mantel-Cox log-rank comparison (**Figure 2A**). Reactivation from the lungs did not differ significantly between ΔM33_stop_ and KP (**Figure 2B**). Interestingly, ΔM33_stop_ reactivated from the salivary glands in a minority of the samples, indicating that a small reservoir of latent virus is present in that tissue despite the absence of detectable replicating virus during acute infection (**Figure 2C**). US28^+^ was found to be less efficient at reactivation than KP, but reactivation could be detected in all tissues and from over half of salivary gland explants (**Figures 2A-C**). After initial reactivation, both US28^+^ and ΔM33_stop_ appear to replicate normally (**Figures 2D-F**).

Previous studies have demonstrated a direct relationship between the viral genome copy number and the rate of reactivation (41, 42). The cumulative probability of a single viral genome to reactivate from latency over the course of an *ex vivo* culture assay has been estimated at 10^-4^ (41). In order to determine whether the latency defect we observed was in fact due to a lower viral genome copy number, DNA was purified from the lungs and spleens of latently-infected mice at 70 dpi, and the genome copy-to-cell ratio was determined by quantitative PCR. Although ΔM33_stop_ had a slightly lower average genome copy number, this was subject to high variability between animals and did not reach the level of statistical significance (p>0.2). A lower latent genome load in ΔM33_stop_-infected animals was therefore not sufficient to explain the observed differences in reactivation (**Figure 2G**).

### M33 alters T cell activation during acute MCMV infection without affecting viral clearance

**Figure 3:**
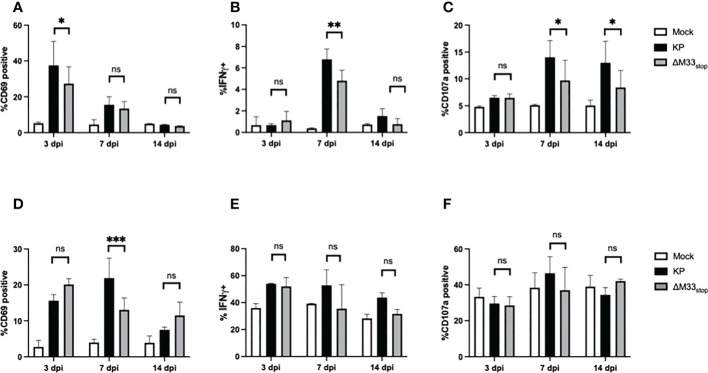
CD8^+^ T cell responses to acute infection with KP and ΔM33_stop._ BALB/c ByJ mice were infected with KP or ΔM33_stop_ and sacrificed at the indicated time points. **(A-C)** CD3^+^CD8^+^ lymphocytes were isolated from the spleen, stained with fluorescent antibodies, and analyzed by flow cytometry. CD69 **(A)**, IFNγ **(B)**, and CD107a **(C)** are shown. **(D-F)** CD3^+^CD8^+^ lymphocytes were isolated from the lung as above. CD69 **(D)**, IFNγ **(E)**, and CD107a **(F)** are shown. Pairwise comparisons are shown only between KP and ΔM33_stop_. Experiments were conducted in duplicate with at least 4 animals per group (n = 8). Significance shown as *, p< 0.05; **, p<0.01; ***, p<0.001; ns, not significant.

T cell responses during latency are distinct from those that occur during acute infection, with a more restricted epitope repertoire, higher expression of maturity and exhaustion markers, and shortened telomeres in inflationary clones (21). However, prior research has demonstrated a correlation between the size of the T cell response during acute infection and the memory inflation population (43). Thus, we could not rule out that the initial T cell response may be required to ‘prime’ memory inflation. Therefore, we first set out to characterize the T cell response to M33-deficient MCMV during acute infection. Female BALB/c ByJ mice were inoculated as above with 1x10^6^ PFU of KP or ΔM33_stop_ MCMV and sacrificed at the indicated time points. Splenocytes and lung T cells were isolated, stained, and analyzed by flow cytometry. In the spleen, numbers and percentages of CD8^+^ T cells and CD4^+^ T cells were similar between groups. Maturity and activation status were assessed using CD62L as a marker of naïve and central memory T cells and CD69 as a marker of early activation and lung tissue residency. Effector function was determined based on IFN-γ and LAMP1 (CD107a) expression, which correspond to antiviral capacity and degranulation status. The inhibitory receptor KLRG1 was also used, both as a marker of antigen experience and as a means of correlating the early T cell response and later memory inflation, which specifically involves CD8^+^KLRG1^+^ T cells. We found that, while a few differences were detectable between KP- and ΔM33_stop_-infected mice at 7 dpi, these differences had resolved by 14 dpi (**Figures 3A–F**). Notably, activation of T cells was significantly increased in the spleens of KP-infected animals at 7 dpi, with higher levels of intracellular IFN-γ and surface CD107a. IFN-γ was no longer significantly different by 14 dpi, while CD107a remained elevated (**Figures 3B, C**). In the lung, a higher percentage of CD8^+^ T cells showed high surface expression of CD69 at 7 dpi in KP-infected compared to ΔM33_stop_-infected animals, but no differences were detected in IFN-γ or CD107a (**Figures 3D-F**). Additionally, no differences were found between ratios of CD8^+^ to CD4^+^ T cells (**Figure S3**) or CD8^+^KLRG1^+^ T cells (**Figure S4**) at any time point. Interestingly, numbers and percentages of active CD8^+^ T cells did not correlate significantly with MCMV titer in infected tissues (**Figure S5**).

### T cells from mice latently infected with ΔM33_stop_ or KP MCMV undergo memory inflation with differential epitope specificity

**Figure 4:**
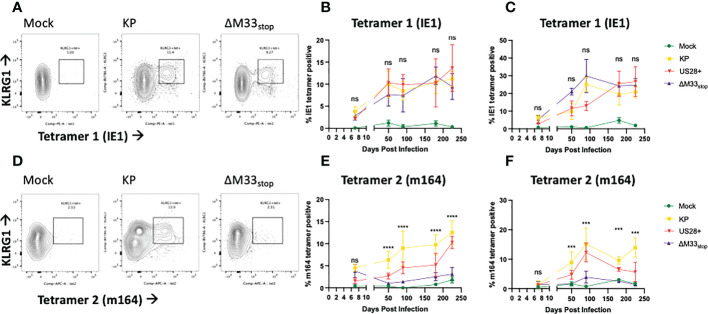
KP and ΔM33_stop_ induce differential epitope-specific T cell memory inflation. BALB/c ByJ mice were infected with KP or ΔM33_stop_ or mock infected and sacrificed at time points from 7 dpi to 225 dpi. Flow cytometry was performed on lung- and spleen-derived lymphocytes, and gated on CD3^+^CD8^+^ cells, representing CTLs. MHC tetramers loaded with peptides AGPPRYSRI (IE1) and YPHFMPTNL (m164) were used to label epitope-specific T cells. Shown are percentages of KLRG1^+^tet^+^ T cells out of total CD8^+^ T cells. **(A, D)** Representative samples from 225 dpi are shown at left to demonstrate gating strategy. **(B-C)** IE1 tetramer-specific T cells in the spleen **(B)** and lung **(C)** from mock (green), KP (yellow), US28^+^ (red) and ΔM33_stop_ (purple) infected mice. **(E-F)** m164 tetramer-specific T cells in the spleen **(E)** and lung **(F)**. Minimum n=5 at each time point, except US28^+^ which is minimum n=3. ANOVA with pairwise comparison between KP and ΔM33_stop_; significance is shown as ***p<0.001; ****p<0.0001; ns, not significant.

The differences in reactivation from latency of ΔM33_stop_ led us to question whether T cell memory inflation would occur in ΔM33_stop_-infected mice as it does in wild-type MCMV-infected mice. During latent MCMV infection of BALB/c mice, T cell clones recognizing IE1 (YPHFMPTNL) and m164 (AGPPRYSRI) peptides form the described inflationary population (27). However, it is not fully understood whether these proteins are expressed during latency or whether they represent stochastic reactivation events in tissue. We hypothesized that M33 would not directly affect transcripts expressed during latency (TELs), which have been previously characterized (22, 24, 25). Instead, we expected the presence or absence of the M33 protein to alter the frequency of reactivation events in tissues. To test this, female BALB/c ByJ mice were inoculated IP with 1x10^6^ PFU of KP, US28^+^, or ΔM33_stop_ MCMV. At intervals from 8 to 225 dpi, mice were sacrificed and flow cytometry was performed on lung- and spleen-derived T cells. As expected, KP induced memory inflation of both IE1- (YPHFMPTNL) and m164- (AGPPRYSRI) specific KLRG1^+^CD8^+^ T cells (**Figures 4A-C**) (27). ΔM33_stop_ induced IE1-specific CD8^+^ T cell memory inflation similar to KP, reaching approximately 10% of total CD8^+^CD62L^-^ T cells in the spleen and over 20% in the lung (**Figures 4A-C**). Strikingly, ΔM33_stop_ caused minimal m164-specific CD8^+^ T cell memory inflation, with no significant difference from the low level of nonspecific tetramer binding observed in mock-infected mice (**Figures 4D-F**). Our results were consistent between replicates, at widely spaced time points, and continuing to at least 225 dpi, indicating that the difference is neither transient nor subject to variability between animals. Interestingly, we found that animals infected with US28^+^ showed similar levels of IE1-specific memory inflation and an intermediate level of m164-specific memory inflation (**Figures 4E, F**). These findings suggest that US28, which is able to complement M33 in certain other functional aspects (32), is additionally able to partially restore the interactions of MCMV with the immune system. Collectively, these data suggest that M33 influences m164 epitope-specific CD8^+^ T cell memory inflation in MCMV-infected BALB/c mice and that US28 partially complements this function.

### Fewer m164 epitope-specific T cells in ΔM33_stop_-infected mice result in diminished cytotoxicity *ex vivo*

The flow cytometry data suggested that we should expect a differential T cell-mediated cytotoxicity response in animals infected with the wild-type and mutant viruses. In order to confirm that the IE1- and m164- specific tetramer staining did in fact correspond to matching T cell functionality, leukocytes were isolated from the spleens of mice acutely or latently infected with KP, ΔM33_stop_, or US28^+^ MCMV. To measure *ex-vivo* cytotoxicity, splenic leukocytes or magnetic activated cell sorting (MACS)-enriched T cells were added to cultures of a salivary gland-derived mesenchymal-like cell line, mSGM, which we previously isolated and described (35). These target cells were selected due to their expression of all 3 isotypes of MHC class I which are found in BALB/c mice. Cytotoxicity was measured in real-time using a non-toxic fluorescent caspase-3/7 substrate, NucView488 (a fluorogenic dye coupled to the caspase DEVD cleavage recognition site) on an Incucyte live-cell imaging system. To selectively present the m164 peptide epitope, a stable lentivirus-transduced cell line, mSGM-m164, was used as previously described (35). Although we attempted to create a stable cell line expressing IE1 using the same lentivirus backbone, we found that IE1-transduced cells failed to proliferate and died off after a few days. To remedy this, we used an adenovirus carrying the IE1 open reading frame, Ad5-MCMV_IE1, to induce transient IE1 expression (35).

**Figure 5:**
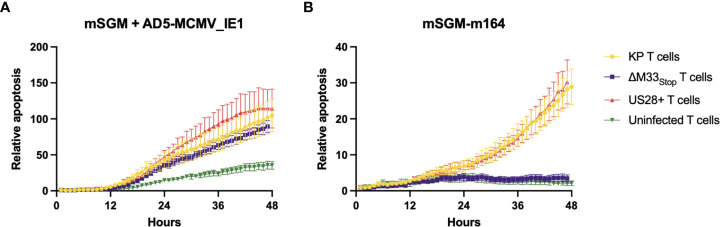
Fewer m164 epitope-specific T cells from mice latently infected with ΔM33_stop_ result in decreased cytotoxicity, and this defect is rescued by US28. MACS-enriched T cells from mice infected with KP (yellow), ΔM33_stop_ (purple), or US28^+^ (red) MCMV or left uninfected (green) were added at an effector:target ratio of 6:1 to mSGM mesenchymal cells infected with Ad5-MCMV_IE1 **(A)** or mSGM-m164 cells **(B)**. Apoptosis is shown as a ratio compared to time 0 and normalized to confluence of the cell monolayer.

To demonstrate the effects of memory inflation, we used T cells from an early time point (30 dpi) and a later time point during latency (60 dpi). As expected, enriched T cells from latently KP-infected mice were able to efficiently kill both IE1- and m164-expressing target cells (**Figures 5A, B**), while sparing both uninfected mSGM cells and Ad5-control-infected mSGM cells (**Figure S7**). In contrast, T cells from latently ΔM33_stop_-infected mice were able to kill IE1-expressing but not m164-expressing target cells (**Figures 5A, B**), corresponding to the lack of m164 tetramer-positive T cells seen in the flow cytometry results. Interestingly, T cells derived from mice latently infected with US28^+^ were fully competent at killing both IE1- and m164-expressing target cells, despite the slightly lower percentage of m164 tetramer-positive T cells detected by flow cytometry (**Figure 5**). In all KP-infected animals, T cells from mice sacrificed at 60 dpi were significantly more competent at killing target cells compared with mice sacrificed at 30 dpi (p<0.0001). T cells from control, mock-infected mice did not exhibit increased cytotoxicity against any of the target cells (**Figure S7**). In all cases, differential rates of apoptosis became apparent after around 12 hours and increased until at least 48 hours after the addition of T cells, at which point experiments were terminated. These results indicate that the absence of m164 tetramer staining in T cells from ΔM33_stop_-infected mice corresponds to an inability to target m164-expressing cells

### ΔM33_stop_ shows delayed gene expression during latency and impaired gene phase transition during reactivation

**Figure 6:**
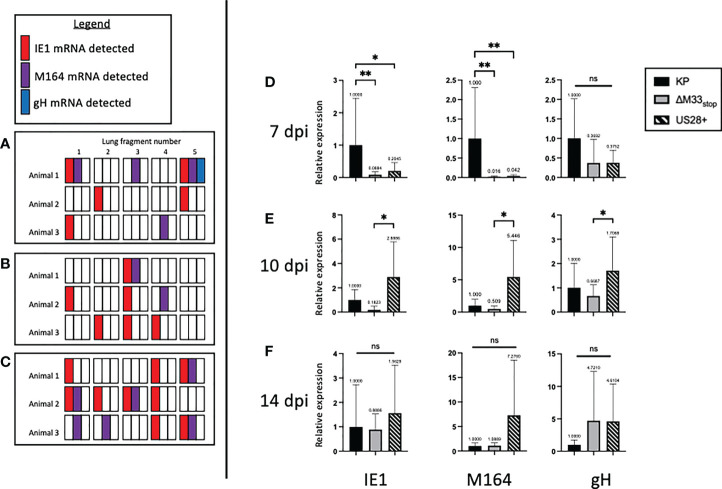
M33 is required for progression to β- and γ-phase genes and US28 partially complements its effects. Latently-infected BALB/c mice were sacrificed at 70 dpi and the lungs were divided into 5 lobes. RNA was purified from lung tissues, and RT-PCR was performed to detect viral RNA transcripts. Each square represents one lobe, and each row of squares represents one animal (n=3). **(A-C)** Presence or absence of IE1 (red), m164 (purple), or gH (blue) mRNA in lung lobes of mice latently infected with KP **(A)**, ΔM33_stop_ **(B)**, or US28^+^ **(C)**. **(D-F)** Lungs from latently-infected mice were explanted and RNA purified at 7 dpe **(D)**, 10 dpe **(E)**, and 14 dpe **(F)**. Data are normalized to KP (black bar) and shown as relative expression compared to GAPDH housekeeping gene. Significance shown as *p<0.05; **p<0.01; ns, not significant. If no comparisons are indicated, no significance was detected. A-C, n=3 mice; D-F, n=2 mice divided into 3 replicate wells each.

Our flow cytometry results suggested a defect in m164 epitope-specific CD8^+^ T cell memory inflation in ΔM33_stop_ -infected mice. One explanation for this could be a delay in progression from α-phase (immediate-early) genes to β-phase (early) and subsequently γ-phase (late) genes during reactivation from latency (44). After determining that CD8^+^ T cell epitope specificity differed in latently ΔM33_stop_ -infected animals, we asked whether ΔM33_stop_ expressed IE1 during latency similar to wild-type MCMV infection (25, 45). However, the m164 ORF, which is transcribed as two isoforms at α- and β-phase time points, might be differentially expressed (46). To test this, we collected lungs of latently-infected mice, divided each lung into 5 lobes, and used quantitative RT-PCR to measure mRNA levels of IE1, m164, and gH. We found that IE1 and m164 were detectable in KP-, US28^+^- and ΔM33_stop_-infected lungs corresponding to their α- and β-phase expression, but gH was detectable only in one KP-infected animal, possibly indicating a reactivation event (**Figures 6A–C**). No significant differences could be detected in expression of any of these genes (p>0.2). As described previously, IE1 transcription did not appear to be essential for m164 transcription to occur (46).

Next, to detect whether gene expression occurs at the same levels during reactivation from latency, we performed tissue explant assays. Rather than culturing for 6 weeks, we collected a fraction (~1/3) of the tissue fragments at 7, 10, and 14 days post explant (dpe), and performed quantitative RT-PCR to detect IE1, m164, and gH, as above. At 7 dpe, when infectious virus remained below the limit of detection by plaque assay (data not shown), KP-infected lungs were positive for all three transcripts. However, ΔM33_stop_ had approximately 50-fold lower gene expression, and US28^+^ showed an intermediate level of expression (**Figure 6D**). By 10 dpe, US28^+^ had reached gene expression levels similar to KP, but ΔM33_stop_ gene expression still lagged slightly (**Figure 6E**). At 14 dpe, gene expression between all three viruses was no longer significantly different (**Figure 6F**). gH expression levels did not differ significantly between viruses and were low in all three viruses, rarely reaching the threshold of detection. These data suggest that, during reactivation, M33 is required for reinitiating gene expression and lytic replication.

### ΔM33_stop_ reactivates in B cell-deficient mice with reduced efficiency

**Figure 7:**
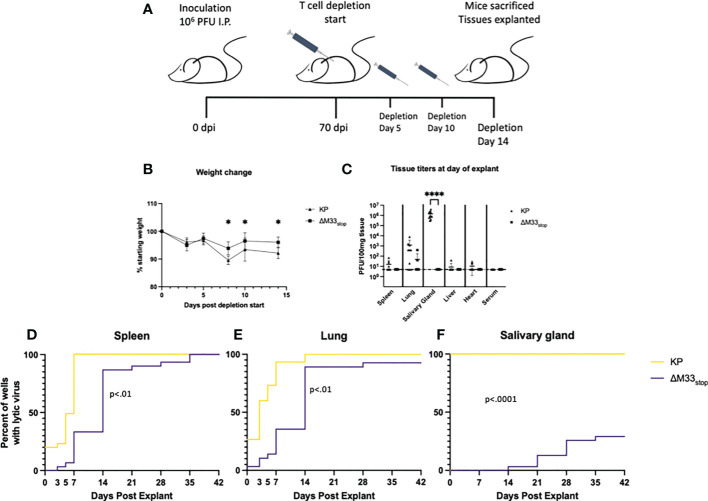
M33 is required for reactivation *in vivo*. Jh mice lacking B cells were inoculated IP with 10^6^ PFU of either KP or ΔM33_stop_. At 70 days post infection, T cell depletion began using cytolytic α-CD4 and α-CD8 antibodies. Mice were injected twice more, at days 5 and 10. 14 days post depletion start, mice were sacrificed and tissues explanted. **(A)** Schematic illustrating this timeline. **(B)** Mice were weighed periodically during T cell depletion, and ΔM33_stop_ was found to induce less weight loss, suggesting a physiological difference during MCMV reactivation. **(C)** Lytic virus was quantified at the time of sacrifice in a small piece of each tissue by plaque assay. **(D-F)** The remaining tissues were explanted and supernatant was collected at intervals and titered. Reactivation in the spleen **(D)**, lung **(E)**, and salivary gland **(F)** are shown. Data represent the percent of wells with lytic virus detectable by plaque assay, with a limit of detection of 10 PFU/ml (n=10 animals per group, totaling 30 wells per tissue). Statistical tests are ANOVA **(B, C)** or Mantel-Cox log-rank **(D-F)**. Significance is shown as *, p<0.05; ****, p<0.0001.

While *ex vivo* reactivation has long been a standard for assessing latency of herpesviruses, it fails to recapitulate the *in vivo* microenvironment. To examine whether M33 is essential for reactivation *in vivo*, we used a T cell depletion strategy in latently infected mice. Reactivation of lytic virus *in vivo* is decreased by the presence of antibodies, although they are not sufficient for complete protection (6). In order to avoid the interference of antibodies, we used an immunoglobulin heavy chain knockout strain of BALB/c mice, *C.Cg-Igh-J^tm1Dhu^* (referred to hereafter as BALB/c Jh), which lacks functional B cells (47). A previous study using a similar immunoglobulin knockout on C57/BL6 background found that depletion of CD8^+^ and CD4^+^ T cells was sufficient to induce reactivation of wild-type MCMV in nearly all animals (6). We first checked whether the BALB/c Jh mouse strain was susceptible to MCMV infection because certain BALB/c sub-strains have proved more resistant to infection in our hands (unpublished data). Mice were inoculated with 1 x 10^6^ PFU of KP MCMV by IP injection and tissue titers were determined at 3 dpi by plaque assay. Titers reached 10^4^ PFU/g tissue in the spleen and liver and 10^3^ PFU/g in the lung, closely matching titers in BALB/c ByJ mice and indicating good susceptibility to MCMV (**Figure S8**). For latency reactivation studies, mice were infected as above with KP or ΔM33_stop_. Latency at 70 dpi was confirmed by explant assay in a subset of mice (**Figure S9**). To induce *in vivo* reactivation of latent MCMV, T cells were depleted from the remaining latently-infected BALB/c Jh mice using cytotoxic antibodies as described in methods (6) (depletion schematic shown in **Figure 7A**). During the course of depletion, weight loss was detected in both groups, but KP-infected mice lost significantly greater percentage of their body weight by 7 days post depletion (**Figure 7B**). Despite weight loss, no animals reached weight loss >20%.

Flow cytometric analysis of splenocytes from mice post-depletion confirmed that a reduction of >98% of CD8^+^ and >95% of CD4^+^ T cells compared to control was achieved (**Figure S10**). At 14 days post depletion, mice were sacrificed and salivary gland, lung, heart, and spleen were collected. A small piece (~10 mg) of each tissue was homogenized, sonicated, and titered on NIH 3T3 fibroblasts to determine whether virus was actively replicating at the time of sacrifice. The remainder of the tissues were evaluated by explant reactivation assay and the culture supernatants were titered at 0, 3, 5, and 7 dpe and every 7 days thereafter.

Following T cell depletion of the infected BALB/c Jh mice, wild-type MCMV had reactivated in 100% of salivary glands, as well as approximately 70% of lungs and 50% of spleens (**Figure 7C**). Replicating virus was detected in the heart of only 1 animal, but given our previous peak titers in this tissue, which never exceeded 10^2^ PFU, this result is not surprising (48). ΔM33_stop_, by contrast, was not detectable in the salivary glands, in agreement with previous studies showing M33 is required for salivary gland replication (30, 33, 34), and was found in only 10% of lungs and spleens, indicating a strong defect in *in vivo* reactivation from latency (**Figure 7C**).

At 7 dpe, 100% of KP-infected tissues had detectable MCMV in the explant reactivation culture supernatants, suggesting that the virus had emerged from latency either prior to sacrifice of the animals or within a few days of explant since latent tissues are negative for detectable virus at 7 dpe. In comparison with KP, ΔM33_stop_ was slower to reach the limit of detection by plaque assay, though most tissues reactivated *in vitro* by 14 dpe (**Figures 7D-F**). Several lung fragments from ΔM33_stop_-infected animals failed to reactivate, in contrast with KP, which reactivated from all tissue fragments. Some *in vitro* reactivation was observed from the salivary glands of ΔM33_stop_-infected mice, with tissue from 4 out of 10 mice reactivating in at least one well. Overall, T cell depletion was not sufficient to induce reactivation *in vivo* in ΔM33_stop_-infected mice. This suggests that the impaired reactivation observed in ΔM33_stop_-infected mice is indeed due to a defect in the transition from latent to lytic infection and not due to control by the adaptive immune system.

### MHC class I isotype expression is altered by M33 and m164

**Figure 8:**
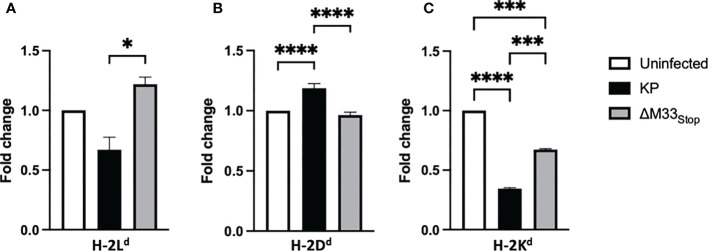
M33 alters MHC expression. mSGM cells were infected at an MOI of 5 with either KP or ΔM33_stop_. 12 hours post infection, cells were stained and analyzed by flow cytometry. Fold change indicates the ratio of cell expressing high levels of H-2L^d^ **(A)**, H-2D^d^ **(B)**, and H-2K^d^ **(C)** compared to uninfected control cells. H-2L^d^ is the MHC isotype responsible for presentation of the IE1 immunodominant peptide and H-2D^d^ is responsible for presenting the m164 immunodominant peptide. Significance is shown as *p<0.05; ***p<0.001; ****p<0.0001. Data represent 3 experiments run in duplicate (total n=6).

A unique feature of the immunodominant IE1 peptide is its presentation pattern on MHC class I. During the α phase of infection, the immunodominant IE1 peptide *YPHFMPTNL* is presented to CTLs on H-2L^d^ -isotype MHC class I. However, during the β phase of infection, immune evasion proteins encoded in the MCMV genome prevent the export of H-2L^d^ from the endoplasmic reticulum, diminishing the presentation of the IE1-derived peptide (49, 50). Despite this immune subversion, infected cells are able to load and export another MHC class I isotype, H-2D^d^, which presents the m164-derived peptide *AGPPRYSRI* (49). These changes in MHC isotype result in differential presentation of peptides from IE1, during α phase only, and m164, during β phase and later. We questioned whether ΔM33_stop_ would differentially regulate MHC expression on infected cells, and if so, whether this difference might contribute to the altered memory inflation phenotype in our studies. To test this, we infected mSGM cells, which express all 3 BALB/c ByJ MHC isotypes (35). After 12 hours of infection with either KP or ΔM33_stop_, MHC expression was assessed by flow cytometry, using antibodies specific for H-2D^d^, H-2K^d^, and H-2L^d^. At 12 hpi, KP was found to downregulate H-2L^d^ and H-2K^d^ while upregulating H-2D^d^. (**Figures 8A-C**). ΔM33_stop_ failed to downregulate H-2L^d^, the isotype which is responsible for presenting the immunodominant IE1 peptide to CD8^+^ T cells, instead resulting in its upregulation (**Figure 8A**). Additionally, ΔM33_stop_-infected cells did not alter expression of H-2D^d^, the isotype which presents the m164 peptide (**Figure 8B**). While ΔM33_stop_-infected cells did downregulate H-2K^d^, it was to a lesser degree than KP-infected cells. These results suggest that M33 is necessary for the downregulation of IE1 peptide-presenting H-2L^d^, which may allow IE1-specific T cells more time to recognize and kill reactivating cells *in vivo.* This could partially explain the epitope-specific T cell memory inflation phenotype observed in ΔM33_stop_-infected mice. Interestingly, H-2K^d^, an MHC class I isotype not known to be associated with inflationary T cells in BALB/c mice, was significantly downregulated by both KP and ΔM33_stop_, though the repercussions of this modulation are not clear.

To further investigate the mechanisms of selective MHC modulation, we next examined the role of the m164 protein. We used mSGM cells stably expressing the m164 protein under the HCMV IE1 promoter to ensure high expression levels, as previously described (mSGM-m164) (35). Flow cytometry was performed using antibodies against all 3 MHC isotypes. Interestingly, we found that the MCMV m164 protein alone is sufficient to alter MHC expression patterns (**Figure S11**). Compared with control-transduced cells, m164-expressing cells had lower levels of H-2L^d^ and higher levels of H-2D^d^. This suggests that, in addition to the previously described immune evasion genes, m164 may act to subvert antigen presentation by altering MHC class I isotype levels. These altered expression patterns suggest that m164 peptide presentation is enhanced while presentation of IE1 peptides is inhibited, thereby altering availability of T cell epitopes during long-term immune responses to MCMV.

## Discussion

The mechanisms of T cell memory inflation during MCMV infection remain only partially understood. The prevailing hypothesis is that some viral proteins are produced during latency and recognized by patrolling CD8^+^ T cells throughout the body, largely supplanting the prior hypothesis that random reactivation events stimulate T cells at foci in tissues, causing localized proliferation of MCMV-specific T cell clones (22). The most probable implication of our results is that stochastic MCMV protein expression during latency fails to capture the entire picture. Instead, IE1 peptide presentation occurs stochastically during viral latency, while presentation of the m164 epitope requires additional steps toward reactivation. IE1 is known to be expressed at low levels by some latently infected cells without triggering a full reactivation (24, 46, 51). The expression of m164 during latency is more ambiguous; it may require de-silencing of the major immediate early promoter (MIEP) region and subsequent production of IE1 and IE3 proteins (52), or it may occur in the absence of MIEP activation (25, 46). Importantly, IE3 is the major transactivator of MCMV replication and its expression is associated with lytic reactivation, whereas IE1 expression does not necessarily correlate with lytic infection (24, 45, 52). This is in contrast with HCMV, in which IE1 is the major transactivator and is considered a hallmark of lytic infection (53–55).

The infection of host cells and establishment of latency are prerequisites for the inflationary T cell memory response, while cell-to-cell spread is not essential (56). Our results may point to another factor contributing to the memory inflation phenotype: the progression of gene class transcription during reactivation. Herpesvirus gene expression is divided into three or four classes: α, β, and γ, corresponding to immediate-early, early, and late genes, with further subdivision of γ genes into “leaky late” and “true late” (44). While both HCMV and MCMV genes can be divided into these classes, MCMV m164 is not clear-cut in its expression class, with detectable RNA transcripts appearing earlier than most β-class genes but with m164-derived peptide presentation occurring with β-class proteins (49). Two distinct m164 transcripts can be detected during latency and reactivation: a short transcript, comprised only of the m164 ORF and expressed after initiation of β-phase gene transcription; and a long transcript, initiated at the upstream m167 reading frame and continuing through m166, m165, and m164, which is detectable during α-phase transcription, albeit at lower levels (25, 46). *In vitro* results suggest that this α-phase transcript is sufficient to activate CTL responses (46). However, whether m164 transcripts *in vivo* correlate with m164 peptide presentation, and if so, whether it is sufficient to induce T cell-mediated cytotoxicity and subsequent memory inflation is unknown.

Our results showing IE1- but not m164-specific CTL memory inflation in ΔM33_stop_-infected mice suggest that MCMV IE1 expression during latency is sufficient to stimulate continuous proliferation of epitope-specific CD8+ T cells, while presentation of m164 antigenic peptides is contingent upon the participation of other viral factors. Transcript-level analysis of gene expression in latently-infected mice suggests that IE1 mRNA transcription occurs in isolation, without the transcription of the IE3 isoform of the same gene locus (45). Whether the transient expression of IE1 alone is indicative of true transcript expressed during latency (TEL) or the incidental detection of a reactivation event which would subsequently be eliminated by CTL responses remains ambiguous. In either case, at least some cells in mice latently infected with wild-type MCMV must progress to the production of m164 proteins in order for peptide presentation to occur, either in isolation or in the presence of IE1. Our results suggest two possibilities: the first is that m164 protein production occurs only in IE1-expressing cells, and that a delay in m164 production is sufficient to allow CTL intervention prior to the presentation of m164 peptides; the second is that some factor after MIEP activation induces apoptosis or aborts reactivation in M33-deficient cells before m164 peptide presentation can occur.

Viral chemokine receptor homologs are pleiotropic proteins that serve as initiators of signaling cascades (29) and bind and sequester host chemokines to suppress immune cell recruitment (57). M78, another viral GPCR homolog, induces the buildup of viral mRNA transcripts to enhance viral gene expression during early infection (58). It is possible that M33 serves a similar function during latency or reactivation. However, our qPCR data show m164 gene expression in the lungs of ΔM33_stop_-infected mice, indicating that its transcription is not contingent on the presence of M33. Combined with the complete absence of m164-specific CTLs, this suggests that the presence of m164 transcripts does not always correlate with peptide presentation.

Manipulation of cellular MHC is a well-known immune evasion mechanism employed by herpesviruses (59). Downregulation of certain MHC class I isotypes, mediated by m04, m06, and m152 genes, may play a role in the differential presentation (60), but studies in C57BL/6 mice demonstrate that these immune evasion genes do not substantially alter T cell memory inflation (61). Nevertheless, many antiviral immune responses vary between mouse strains, and we cannot rule out the possibility that M33 interacts with one or more of these proteins, or increases their expression during reactivation, thereby altering the BALB/c strain immune response. Although m04, m06, and m152 are the only MCMV genes which have been described to alter peptide presentation on MHC (49), our data using the m164-expressing mSGM cell line indicate that a similar role may be ascribed to the m164 protein, perhaps leading to the preferential presentation of m164- rather than IE1-derived peptides (**Figure S11**). A delay in expression of m164 in ΔM33_stop_-infected cells during latent infection *in vivo* may therefore be responsible for the differential immune response. Further research on HCMV proteins which may play similar roles in modulating the immune response is certainly warranted.

Our results indicate that the T cell memory inflation observed in MCMV-infected mice may not be easily categorized as requiring reactivation or simply TELs. Instead, T cell responses may be modulated by other factors, including the viral GPCRs M33 and US28. Our new insights into the increased rate of apoptosis of ΔM33_stop_-infected cells and the altered MHC isotype expression on the cell surface suggest new roles for viral GPCRs in immune evasion. Finally, our assessment of reactivation of ΔM33_stop_ from T cell- and immunoglobulin-deficient mice demonstrates further roles for M33 in latency and reactivation, emphasizing the need for more study of viral GPCRs and their involvement in the pathogenesis of HCMV disease.

Collectively, our results demonstrate that the M33 GPCR homolog of MCMV plays roles in multiple aspects of immune evasion, altering T cell memory inflation and MHC isotype expression. We further confirm its role during reactivation from latency, even in the absence of antibodies and T cell immune pressure. Notably, our results point to novel, pleiotropic functions of a viral GPCR homolog, which may lead to a better understanding of viral immune evasion strategies and possible therapeutic approaches to treating latent cytomegalovirus infection in humans.

## Data availability statement

The raw data supporting the conclusions of this article will be made available by the authors, without undue reservation.

## Ethics statement

The animal study was reviewed and approved by Louisiana State University School of Veterinary Medicine Institutional Animal Care and Use Committee.

## Author contributions

Studies were designed by TW, RC, and BS. Experiments were carried out by TW, BS, and CB. Mutant viruses were provided by HF and ND-P. Manuscript was written by TW and RC and edited by TW, RC, BS, HF, ND-P, and CB. Funding for the study was provided through the USDA NBAF Scientist Training Program fellowship awarded to TW and the Louisiana State University School of Veterinary Medicine startup fund awarded to RC. All authors contributed to the article and approved the submitted version.

## Supplementary material

Supplementary Figure 1 The ΔM33_stop_ M33 sequence contains 3 in-frame stop codons. The M33 gene locus was PCR amplified and Sanger sequenced at the stop codon insertion site. Reference sequence (NCBI:txid10366; top row) alignment with ΔM33_stop_ sequence (bottom row) showing stop codons (*).

Supplementary Figure 2 The AGPPRYSRI immunodominant epitope is intact in ΔM33_stop_ MCMV. The m164 gene locus including the AGPPRYSRI epitope sequence from KP and ΔM33_stop_ MCMV was sequenced by Sanger sequencing and aligned with template sequence from the NCBI database (NCBI:txid10366). Both KP and ΔM33_stop_ produce an intact AGPPRYSRI amino acid sequence. The single-nucleotide difference from the reference (highlighted in red) does not alter the amino acid sequence.

Supplementary Figure 3 Ratios of CD4^+^ to CD8^+^ T cells during acute infection are similar between ΔM33_stop_- and KP-infected animals. The CD4:CD8 ratio is a simple metric of T cell expansion during viral infection, decreasing as CD8^+^ T cells proliferate. In the spleen **(A)** and the lung **(B)** there was no significant difference between in CD4:CD8 ratios during ΔM33_stop_ and KP infection. Both viruses significantly altered the baseline CD4:CD8 ratio. Data represent results of three experiments (total n = 9 per group). Significance is shown as *, p<0.05; **, p<0.01; ***, p<0.001.

Supplementary Figure 4 M33 does not significantly alter KLRG1 expression during acute infection. CD8^+^ T cells were analyzed by flow cytometry for surface expression of KLRG1, a marker of antigen experience. In both the spleen **(A)** and lung **(B)**, KLRG1 increased due to MCMV infection. However, no significant differences were detected between ΔM33_stop_ and KP-infected animals. Data represent results of three experiments (total n = 12 per group). Significance is shown as ***, p<0.001; ****, p<0.0001.

Supplementary Figure 5 Virus titer in the spleen does not correlate with T cell activation. CD69 **(A)** and IFN-γ **(B)** of splenic T cells from KP-infected mice at 7 dpi were quantified by flow cytometry. A portion of each spleen was titered by plaque assay and the resulting titer was used as the independent variable for simple linear regression analysis. The slopes of the lines were not found to differ significantly from zero (p>0.1). A similar analysis carried out with lung T cells from KP-infected mice, and with both splenic and lung T cells from ΔM33_stop_ -infected animals, also failed to find significance (data not shown).

Supplementary Figure 6 Gating strategy for CD8^+^ T cells. Splenocytes were stained with various fluorochrome-tagged antibodies and processed on a flow cytometer. Data were then analyzed using FlowJo software (Beckton Dickinson). Representative image shows gating strategy for markers used for characterization.

Supplementary Figure 7 Enriched T cells from latently MCMV-infected mice kill target cells in an epitope-specific manner. mSGM cells infected with Ad5-control **(A)** were not killed by enriched T cells from uninfected (violet) or latently KP (blue) ΔM33_stop_ (green) or US28+ (yellow) -infected mice at 60 dpi. mSGM cells infected with Ad5-MCMV_IE1 were killed by T cells from all latently-infected animals **(B)**. mSGM cells alone **(C)** were not killed by T cells from any animals. mSGM-m164 cells expressing the MCMV m164 protein were killed by T cells from mice latently infected with KP and US28+, but not ΔM33_stop_. Data represent 2 fields of view from each of 8 wells, and T cells from a minimum of 2 animals (total n=16).

Supplementary Figure 8 MCMV replicates normally in Jh mice during early acute infection. Mice inoculated IP with 1x10^6^ pfu were sacrificed at 3 dpi and tissues were titered. Data represent 2 biological replicates and 3 technical replicates each.

Supplementary Figure 9 MCMV establishes latency in Jh mice. Latency in KP-infected Jh mice at 70 dpi was confirmed by explant assay. Faster MCMV reactivation than seen in wild-type BALB/c ByJ mice is likely due to the lack of neutralizing antibodies.

Supplementary Figure 10 T cell depletion from Jh mice was successful. Flow cytometry was conducted on splenocytes to confirm successful T cell depletion. Undepleted **(A)** and T cell depleted **(B)** splenocytes labeled with αCD8-BV711 (y-axis) and αCD4-PerCP-Cy5.5 (x-axis) are shown to demonstrate depletion of both T cell subsets.

Supplementary Figure 11 Class I MHC expression is altered in m164-expressing cells. Mean fluorescence intensity (MFI, arbitrary units) of H-2D^d^ and H-2L^d^ in mSGM cells (black bars) and mSGM-m164 cells (gray bars). H-2D^d^, which presents the m164 immunodominant peptide, is increased significantly in mSGM-m164 cells compared to control (p<0.001). H-2L^d^, which presents the IE1 immunodominant peptide, is not significantly different between cells expressing m164 and control (p>0.05).

Supplementary Table 1 Virus mutants used in studies.

Supplementary Table 2 Primers used for PCR.
