# Supplementary figures and images for: The CMV-encoded G protein-coupled receptors M33 and US28 play pleiotropic roles in immune evasion and alter host T cell responses
Source: Front Immunol. 2022 Dec 7;13:1047299. doi: 10.3389/fimmu.2022.1047299 (PMC9768342; doi:10.3389/fimmu.2022.1047299)

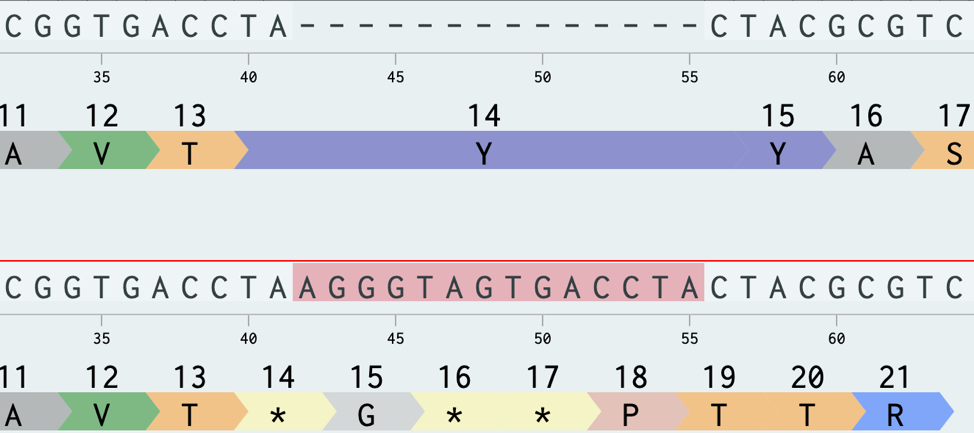

Supplement: Supplementary Figure 1 — The ΔM33stop M33 sequence contains 3 in-frame stop codons. The M33 gene locus was PCR amplified and Sanger sequenced at the stop codon insertion site. Reference sequence (NCBI:txid10366; top row) alignment with ΔM33stop sequence (bottom row) showing stop codons (*). [file Image_1.tif]

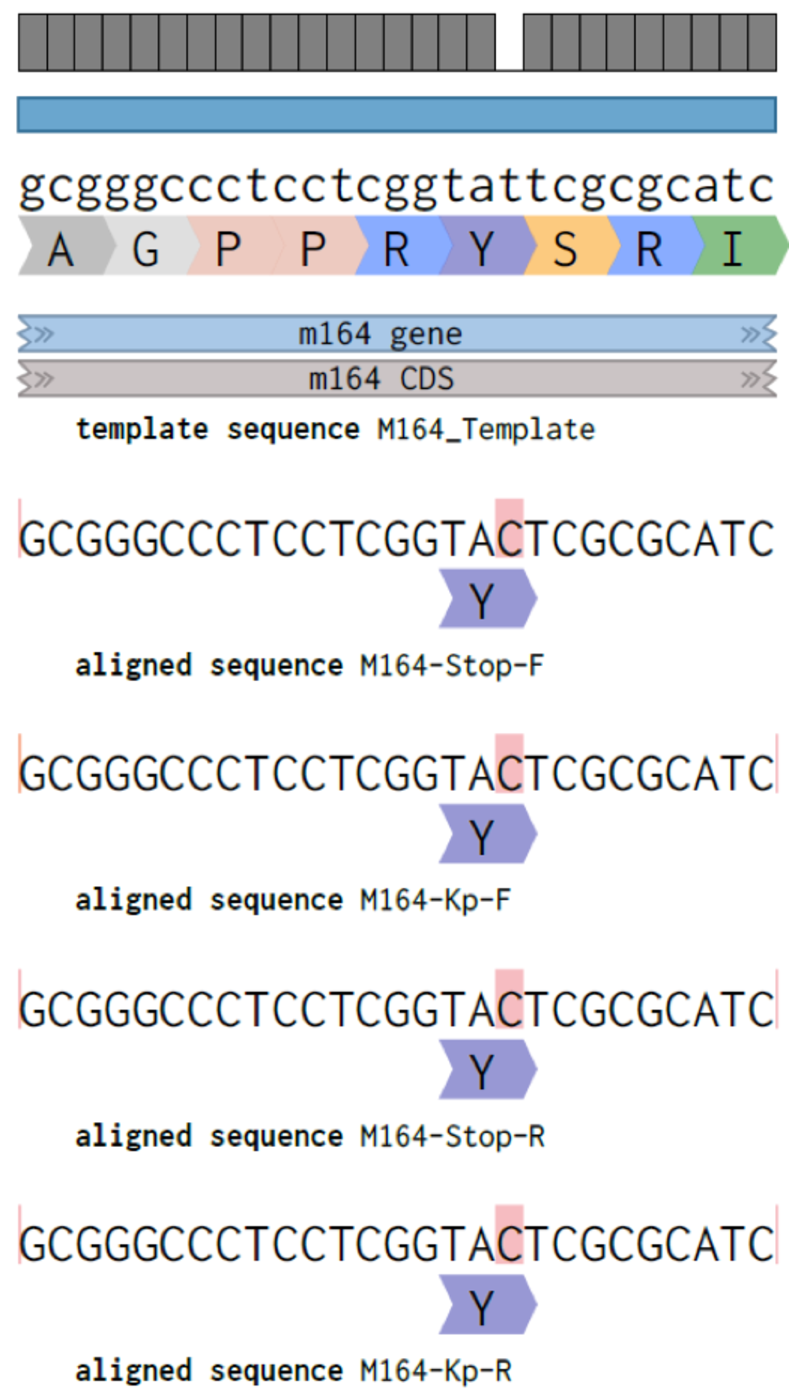

Supplement: Supplementary Figure 2 — The AGPPRYSRI immunodominant epitope is intact in ΔM33stop MCMV. The m164 gene locus including the AGPPRYSRI epitope sequence from KP and ΔM33stop MCMV was sequenced by Sanger sequencing and aligned with template sequence from the NCBI database (NCBI:txid10366). Both KP and ΔM33stop produce an intact AGPPRYSRI amino acid sequence. The single-nucleotide difference from the reference (highlighted in red) does not alter the amino acid sequence. [file Image_2.tif]

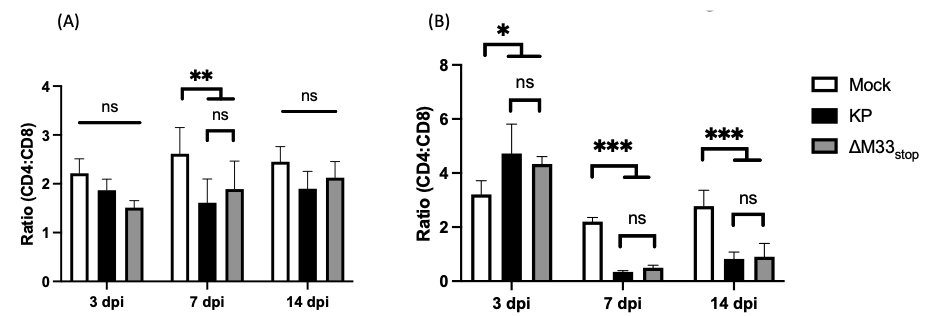

Supplement: Supplementary Figure 3 — Ratios of CD4+ to CD8+ T cells during acute infection are similar between ΔM33stop- and KP-infected animals. The CD4:CD8 ratio is a simple metric of T cell expansion during viral infection, decreasing as CD8+ T cells proliferate. In the spleen (A) and the lung (B) there was no significant difference between in CD4:CD8 ratios during ΔM33stop and KP infection. Both viruses significantly altered the baseline CD4:CD8 ratio. Data represent results of three experiments (total n = 9 per group). Significance is shown as *, p<0.05; **, p<0.01; ***, p<0.001. [file Image_3.tif]

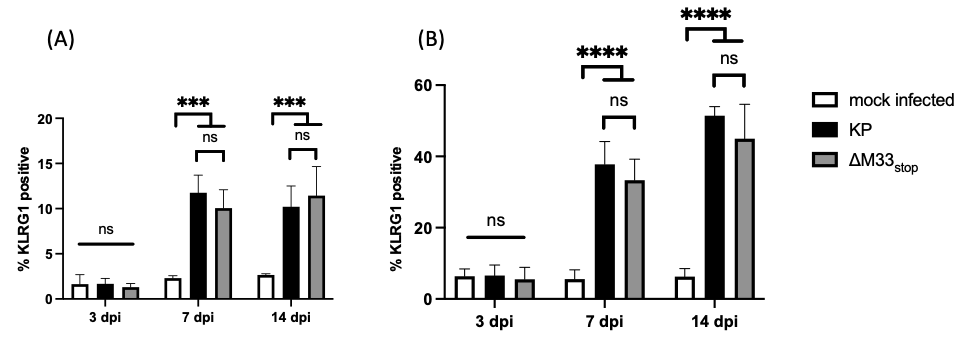

Supplement: Supplementary Figure 4 — M33 does not significantly alter KLRG1 expression during acute infection. CD8+ T cells were analyzed by flow cytometry for surface expression of KLRG1, a marker of antigen experience. In both the spleen (A) and lung (B), KLRG1 increased due to MCMV infection. However, no significant differences were detected between ΔM33stop and KP-infected animals. Data represent results of three experiments (total n = 12 per group). Significance is shown as ***, p<0.001; ****, p<0.0001. [file Image_4.tif]

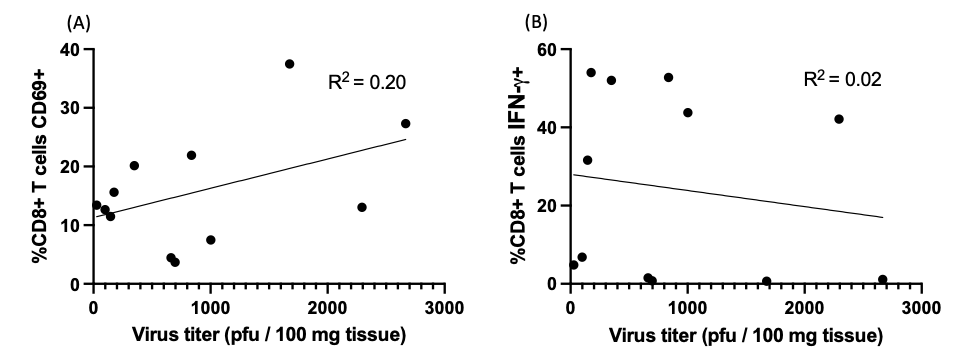

Supplement: Supplementary Figure 5 — Virus titer in the spleen does not correlate with T cell activation. CD69 (A) and IFN-γ (B) of splenic T cells from KP-infected mice at 7 dpi were quantified by flow cytometry. A portion of each spleen was titered by plaque assay and the resulting titer was used as the independent variable for simple linear regression analysis. The slopes of the lines were not found to differ significantly from zero (p>0.1). A similar analysis carried out with lung T cells from KP-infected mice, and with both splenic and lung T cells from ΔM33stop -infected animals, also failed to find significance (data not shown). [file Image_5.tif]

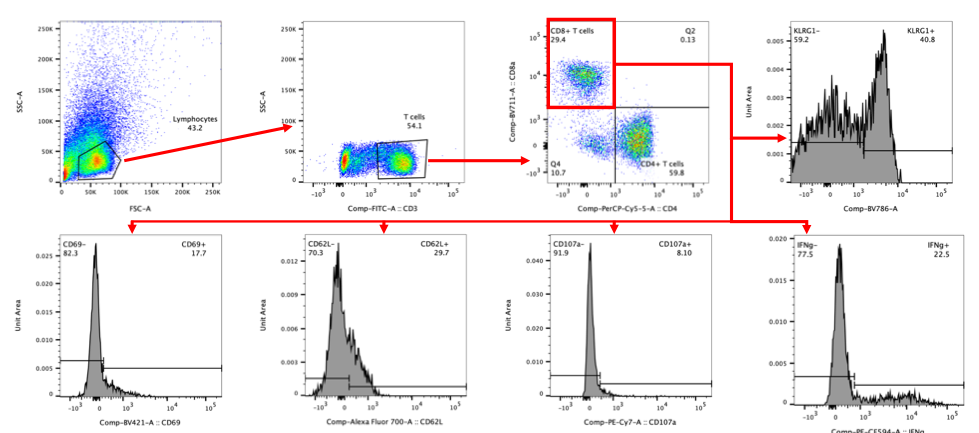

Supplement: Supplementary Figure 6 — Gating strategy for CD8+ T cells. Splenocytes were stained with various fluorochrome-tagged antibodies and processed on a flow cytometer. Data were then analyzed using FlowJo software (Beckton Dickinson). Representative image shows gating strategy for markers used for characterization. [file Image_6.tif]

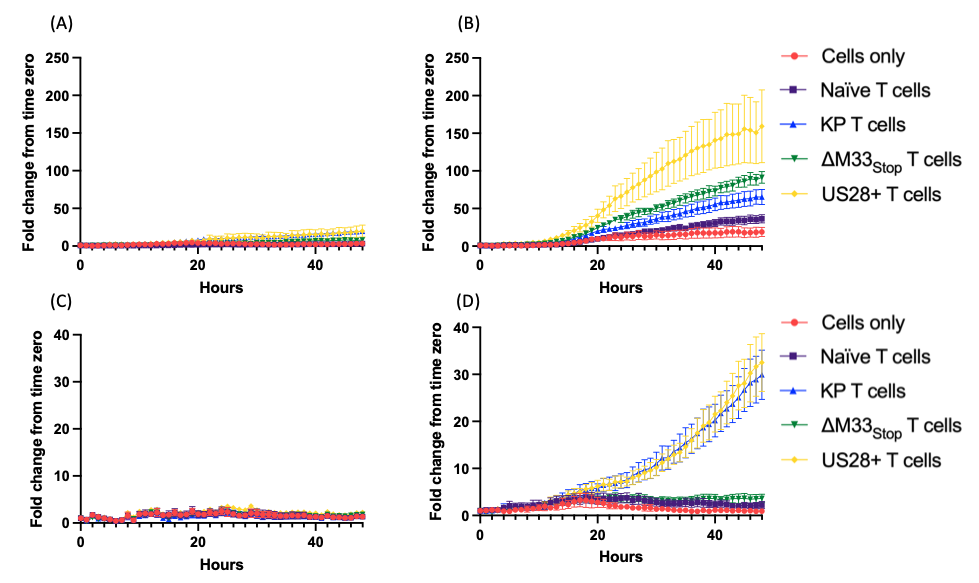

Supplement: Supplementary Figure 7 — Enriched T cells from latently MCMV-infected mice kill target cells in an epitope-specific manner. mSGM cells infected with Ad5-control (A) were not killed by enriched T cells from uninfected (violet) or latently KP (blue) ΔM33stop (green) or US28+ (yellow) -infected mice at 60 dpi. mSGM cells infected with Ad5-MCMV_IE1 were killed by T cells from all latently-infected animals (B). mSGM cells alone (C) were not killed by T cells from any animals. mSGM-m164 cells expressing the MCMV m164 protein were killed by T cells from mice latently infected with KP and US28+, but not ΔM33stop. Data represent 2 fields of view from each of 8 wells, and T cells from a minimum of 2 animals (total n=16). [file Image_7.tif]

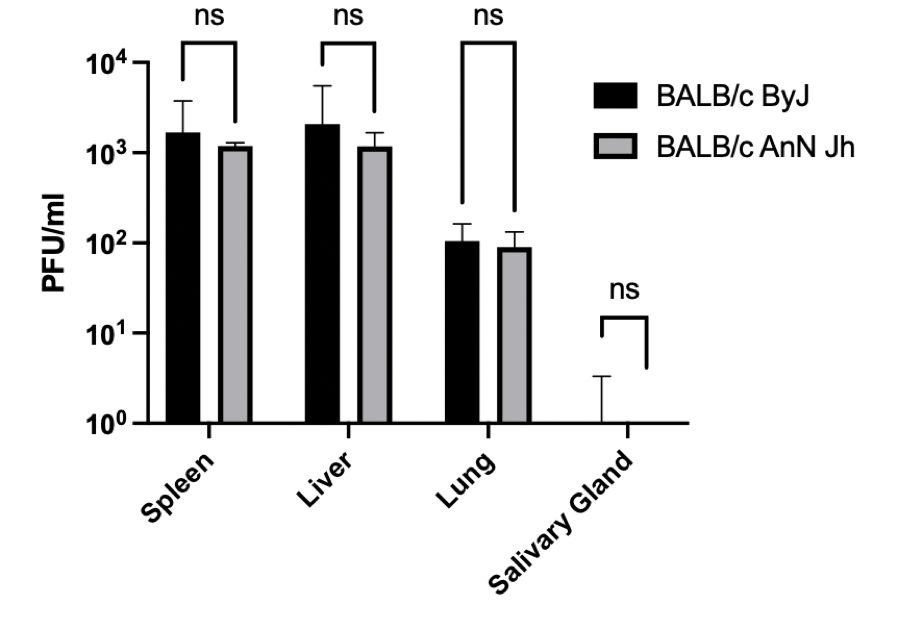

Supplement: Supplementary Figure 8 — MCMV replicates normally in Jh mice during early acute infection. Mice inoculated IP with 1x10^6^ pfu were sacrificed at 3 dpi and tissues were titered. Data represent 2 biological replicates and 3 technical replicates each. [file Image_8.tif]

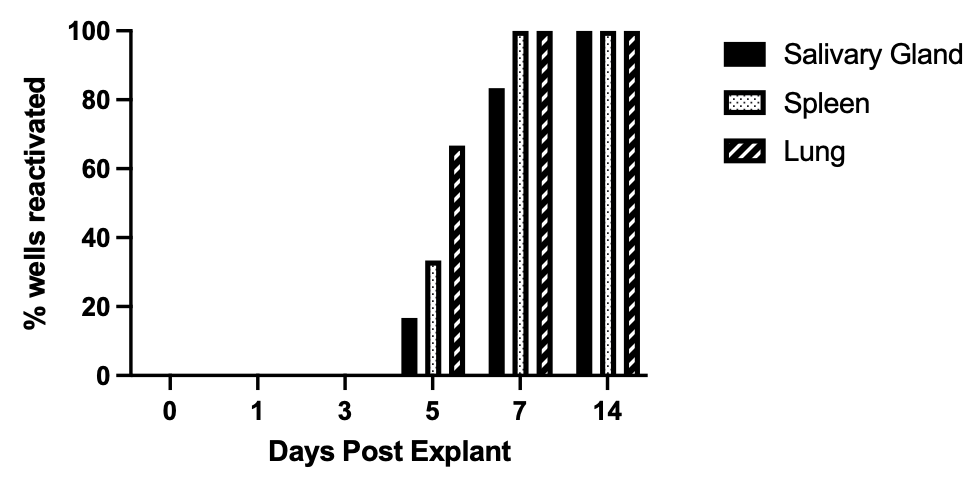

Supplement: Supplementary Figure 9 — MCMV establishes latency in Jh mice. Latency in KP-infected Jh mice at 70 dpi was confirmed by explant assay. Faster MCMV reactivation than seen in wild-type BALB/c ByJ mice is likely due to the lack of neutralizing antibodies. [file Image_9.tif]

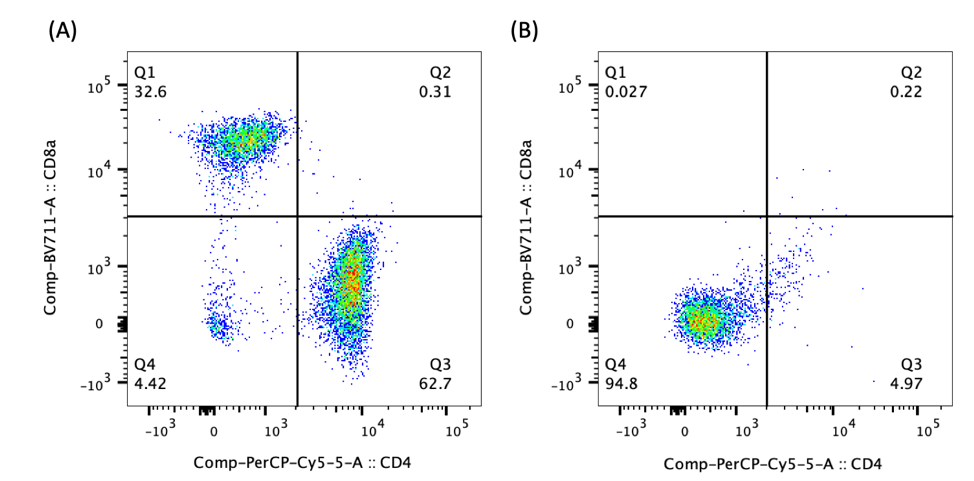

Supplement: Supplementary Figure 10 — T cell depletion from Jh mice was successful. Flow cytometry was conducted on splenocytes to confirm successful T cell depletion. Undepleted (A) and T cell depleted (B) splenocytes labeled with αCD8-BV711 (y-axis) and αCD4-PerCP-Cy5.5 (x-axis) are shown to demonstrate depletion of both T cell subsets. [file Image_10.tif]

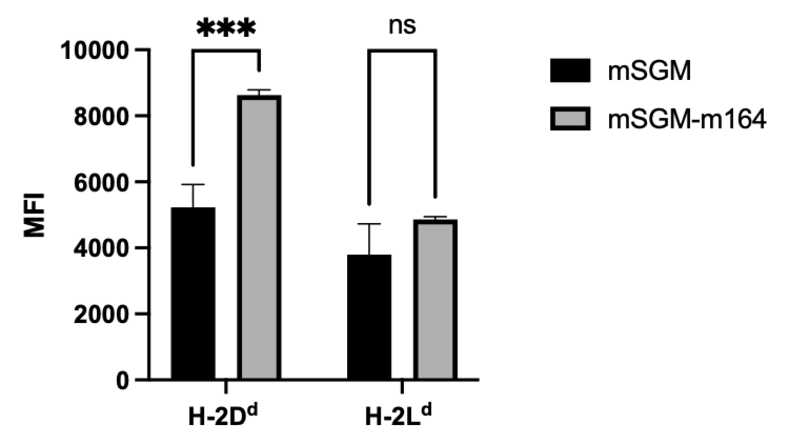

Supplement: Supplementary Figure 11 — Class I MHC expression is altered in m164-expressing cells. Mean fluorescence intensity (MFI, arbitrary units) of H-2Dd and H-2Ld in mSGM cells (black bars) and mSGM-m164 cells (gray bars). H-2Dd, which presents the m164 immunodominant peptide, is increased significantly in mSGM-m164 cells compared to control (p<0.001). H-2Ld, which presents the IE1 immunodominant peptide, is not significantly different between cells expressing m164 and control (p>0.05). [file Image_11.tif]
