# Supplementary material for: The CMV-encoded G protein-coupled receptors M33 and US28 play pleiotropic roles in immune evasion and alter host T cell responses
Source: Front Immunol. 2022 Dec 7;13:1047299. doi: 10.3389/fimmu.2022.1047299 (PMC9768342; doi:10.3389/fimmu.2022.1047299)
Supplement: Supplementary Table 1 — Virus mutants used in studies. [file Table_1.docx]

| **Virus** | **M33 sequence** | **Other gene product** |
| --- | --- | --- |
| K181-Perth (referred to as KP) | Intact | n/a |
| RM427^+^ [63] | Intact | *E. coli* β-galactosidase inserted at the non-essential IE2 ORF |
| ΔM33_stop_ [32] | Disrupted by premature stop codon | none |
| ΔM33_BT2_ [29] | Disrupted by *LacZ* gene | *E. coli* β-galactosidase |
| ΔM33-US28^+^ (referred to as US28^+^) [32] | Removed, replaced with HCMV US28 | HCMV US28 |

**Supplemental Table S1.** **Virus mutants used in studies.**
