# Supplementary material for: The CMV-encoded G protein-coupled receptors M33 and US28 play pleiotropic roles in immune evasion and alter host T cell responses
Source: Front Immunol. 2022 Dec 7;13:1047299. doi: 10.3389/fimmu.2022.1047299 (PMC9768342; doi:10.3389/fimmu.2022.1047299)
Supplement: Supplementary Table 2 — Primers used for PCR. [file Table_2.docx]

| **Gene target** | **Primers** |
| --- | --- |
| Mouse glyceraldehyde-3-phosphate dehydrogenase (GAPDH) | Forward, 5’-CTCACAATTTCCATCCCAGAC-3’  Reverse, 5’-TTTTTGGGTGCAGCGAAC-3’ |
| MCMV early 1 (E1) [64] | Forward, 5’-TCGCCCATCGTTTCGAGA-3’  Reverse, 5’-TCTCGTAGGTCCACTGACGGA -3’ |
| MCMV immediate early 1 (IE1) | Forward, 5’-CCGCACCCAGTTGCAACATGAT-3’  Reverse, 5’-ACGGTTTCGGTTCCAGACTCGA-3’ |
| MCMV m164 | Forward, 5’-CGACGGAGTCCTCTCAGATAA-3’  Reverse, 5’-CAGCTCCCGACAAACTTCTT-3’ |
| MCMV glycoprotein H (gH) | Forward, 5’-CGACGGAGTCCTCTCAGATAA-3’  Reverse, 5’-CAGCTCCCGACAAACTTCTT-3’ |
| MCMV M33 | Forward, 5’-ACGCCGTAAGTCTTCTTCTTC-3’  Reverse, 5’-CACAACATATCTCGCCCTCTC-3’ |

**Supplemental Table S2. Primers used for PCR.**
